# Supplementary material for: Spiritual boredom is associated with over- and underchallenge, lack of value, and reduced motivation
Source: Commun Psychol. 2025 Mar 5;3:35. doi: 10.1038/s44271-025-00216-7 (PMC11882887; doi:10.1038/s44271-025-00216-7)
Supplement: Supplementary file 2 — Supplemental Material [file 44271_2025_216_MOESM2_ESM.pdf]

Online Supplemental Material

SM1_1: *Yoga Boredom Scale – Trait (YBS-T)* 3
SM1_2: *Yoga Boredom Scale – State (YBS-S)* 4
SM1_3: *Meditation Boredom Scale – Trait (MBS-T)* 5
SM1_4: *Meditation Boredom Scale – State (MBS-S)* 6
SM1_5: *Silence Retreat Boredom Scale – Trait (SRBS-Trait)* 7
SM1_6: *Silence Retreat Boredom Scale – State (SRBS-State)* 8
SM1_7: *Sermon Boredom Scale – Trait (SBS-T)* 9
SM1_8: *Sermon Boredom Scale – State (SBS-S)* 10
SM1_9: *Pilgrimage Boredom Scale – Trait (PBS-Trait)* 11
SM1_10: *Pilgrimage Boredom Scale – State (PBS-State)* 12
SM1_11: *Multidimensional State Boredom Scale (MSBS)* 13
SM2_1: *Overchallenge Assessment – Study 1 (Yoga-Trait)* 14
SM2_2: *Underchallenge Assessment – Study 1 (Yoga-Trait)* 15
SM2_3: *Overchallenge Assessment – Study 2 (Yoga-State)* 16
SM2_4: *Underchallenge Assessment – Study 2 (Yoga-State)* 17
SM2_5: *Overchallenge Assessment – Study 3 (Meditation-Trait)* 18
SM2_6: *Underchallenge Assessment – Study 3 (Meditation-Trait)* 19
SM2_7: *Overchallenge Assessment – Study 4 (Meditation-State)* 20
SM2_8: *Underchallenge Assessment – Study 4 (Meditation-State)* 21
SM2_9: *Overchallenge Assessment – Study 5 (Silence Retreat-Trait)* 22
SM2_10: *Underchallenge Assessment – Study 5 (Silence Retreat-Trait)* 23
SM2_11: *Overchallenge Assessment – Study 6 (Silence Retreat-State)* 24
SM2_12: *Underchallenge Assessment – Study 6 (Silence Retreat-State)* 25
SM2_13: *Overchallenge Assessment – Study 7 (Sermon-Trait)* 26
SM2_14: *Underchallenge Assessment – Study 7 (Sermon-Trait)* 27
SM2_15: *Overchallenge Assessment – Study 8 (Sermon-State)* 28
SM2_16: *Assessment – Study 8 (Sermon-State)* 29
SM2_17: *Overchallenge Assessment – Study 9 (Pilgrimage-Trait)* 30
SM2_18: *Underchallenge Assessment – Study 9 (Pilgrimage-Trait)* 31
SM2_19: *Overchallenge Assessment – Study 10 (Pilgrimage-State)* 32
SM2_20: *Underchallenge Assessment – Study 10 (Pilgrimage-State)* 33
SM3_1: *Value Assessment – Study 1 (Yoga-Trait)* 34
SM3_2: *Value Assessment – Study 2 (Yoga-State)* 35
SM3_3: *Value Assessment – Study 3 (Meditation-Trait)* 36
SM3_4: *Value Assessment – Study 4 (Meditation-State)* 37

SM3_5: *Value Assessment – Study 5 (Silence Retreat-Trait)* ... 38
SM3_6: *Value Assessment – Study 6 (Silence Retreat-State)* ... 39
SM3_7: *Value Assessment – Study 7 (Sermon-Trait)* ... 40
SM3_8: *Value Assessment – Study 8 (Sermon-State)* ... 41
SM3_9: *Value Assessment – Study 9 (Pilgrimage-Trait)* ... 42
SM3_10: *Value Assessment – Study 10 (Pilgrimage-State)* ... 43
SM4_1: *Motivation Assessment – Study 1 (Yoga-Trait)* ... 44
SM4_2: *Motivation Assessment – Study 2 (Yoga-State)* ... 45
SM4_3: *Motivation Assessment – Study 3 (Meditation-Trait)* ... 46
SM4_4: *Motivation Assessment – Study 4 (Meditation-State)* ... 47
SM4_5: *Motivation Assessment – Study 5 (Silence Retreat-Trait)* ... 48
SM4_6: *Motivation Assessment – Study 6 (Silence Retreat-State)* ... 49
SM4_7: *Motivation Assessment – Study 7 (Sermon-Trait)* ... 50
SM4_8: *Motivation Assessment – Study 8 (Sermon-State)* ... 51
SM4_9: *Motivation Assessment – Study 9 (Pilgrimage-Trait)* ... 52
SM4_10: *Motivation Assessment – Study 10 (Pilgrimage-State)* ... 53
SM5: *Percentages of Boredom Single-Item Indicator and Scale Values Above Given Thresholds* ... 54
SM6_1: *Correlations Among Variables – Study 1 (Yoga-Trait)* ... 55
SM6_2: *Correlations Among Variables – Study 2 (Yoga-State)* ... 56
SM6_3: *Correlations Among Variables – Study 3 (Meditation-Trait)* ... 57
SM6_4: *Correlations among variables – Study 4 (Meditation-State)* ... 58
SM6_5: *Correlations among variables – Study 5 (Silence retreat - Trait)* ... 59
SM6_6: *Correlations Among Variables – Study 6 (Silence Retreat-State)* ... 60
SM6_7: *Correlations Among Variables – Study 7 (Sermon-Trait)* ... 61
SM6_8: *Correlations Among Variables – Study 8 (Sermon-State)* ... 62
SM6_9: *Correlations Among Variables – Study 9 (Pilgrimage-Trait)* ... 63
SM6_10: *Correlations Among Variables – Study 10 (Pilgrimage-State)* ... 64
SM7_1: *Single-Mean Meta-Analyses of Spiritual Boredom Across Studies – Scale Means* ... 65
SM7_2: *Single-Mean Meta-Analyses of Spiritual Boredom Across Studies – Single-Item Means* ... 66
SM7_3: *Mixed-Effects Meta-Analyses of Correlations with Spiritual Boredom, Moderation by Trait vs. State* ... 67
SM7_4: *Meta-Analyses of Correlations with Spiritual Boredom, Subgroups by Trait vs. State* ... 68
SM8: *Descriptive Statistics on Participants' Current Spiritual Practices* ... 69

SM1: Spiritual Boredom Scales

The German version of the items was used in this study. The English version has been translated via artificial intelligence (deepl.com) and has not yet been psychometrically evaluated.

SM1_1: ***Yoga Boredom Scale – Trait (YBS-T)***

Nr.	English	German
1	When I practice yoga, I usually get bored.	Bei der Ausübung von Yoga langweile ich mich üblicherweise.
2	The physical exercises in yoga usually bore me	Die Körperübungen im Yoga langweilen mich üblicherweise
3	The breathing exercises in yoga usually bore me.	Die Atemübungen im Yoga langweilen mich üblicherweise.
4	The meditation in yoga usually bores me.	Die Meditation im Yoga langweilt mich üblicherweise.
5	The relaxation phase in yoga usually bores me.	Die Entspannungsphase im Yoga langweilt mich üblicherweise.
6	The yoga teacher usually bores me.	Der/Die Yogalehrer*in langweilt mich üblicherweise.
7	The repetitive yoga practice usually bores me.	Der gleichbleibende Ablauf der Yoga-Praxis langweilt mich üblicherweise.

Note. Answer format for the English items ranges from 1 (*completely disagree*) to 5 (*completely agree*). Answer format for the German items ranges from 1 (*stimmt gar nicht*) to 5 (*stimmt genau*).

SM1_2: ***Yoga Boredom Scale – State (YBS-S)***

Nr.	English	German
	During the yoga session I had just completed...	Bei der gerade absolvierten Yoga-Einheit…
1	I was bored.	langeweilte ich mich.
2	I was bored with the physical exercises.	langweilten mich die Körperübungen.
3	I was bored with the breathing exercises.	langweilten mich die Atemübungen.
4	I was bored with the meditation.	langweilte mich die Meditation.
5	I was bored with the relaxation phase.	langweilte mich die Entspannungsphase.
6	I was bored with the yoga teacher.	langweilte mich der/die Yogalehrer/in.
7	I was bored with the repetitive practice.	langweilte mich der gleichbleibende Ablauf.

Note. Answer format for the English items ranges from 1 (*completely disagree*) to 5 (*completely agree*). Answer format for the German items ranges from 1 (*stimmt gar nicht*) to 5 (*stimmt genau*).

SM1_3: ***Meditation Boredom Scale – Trait (MBS-T)***

Nr.	English	German
1	When I practice meditation, I usually get bored.	Bei der Ausübung von Meditation langweile ich mich üblicherweise.
2	The concentration exercises in meditation usually bore me.	Die Konzentrationsübungen in der Meditation langweilen mich üblicherweise.
3	The breathing exercises in meditation usually bore me.	Die Atemübungen in der Meditation langweilen mich üblicherweise.
4	The relaxation phase of meditation usually bores me.	Die Entspannungsphase in der Meditation langweilt mich üblicherweise.
5	The meditation teacher usually bores me.	Der/Die Meditationlehrer*in langweilt mich üblicherweise.
6	The repetitive process of meditation usually bores me.	Der gleichbleibende Ablauf der Meditation langweilt mich üblicherweise.

Note. Answer format for the English items ranges from 1 (*completely disagree*) to 5 (*completely agree*). Answer format for the German items ranges from 1 (*stimmt gar nicht*) to 5 (*stimmt genau*).

SM1_4: ***Meditation Boredom Scale – State (MBS-S)***

Nr.	English	German
	During the meditation session I had just completed...	Bei der gerade absolvierten Meditationseinheit…
1	I was bored.	langeweilte ich mich.
2	I was bored with the concentration exercises.	langweilten mich die Konzentrationsübungen.
3	I was bored with the breathing exercises.	langweilten mich die Atemübungen.
4	I was bored by the relaxation phase.	langweilte mich die Entspannungsphase.
5	I was bored by the meditation teacher.	langweilte mich der/die Meditationslehrer*in.
6	I was bored by the repetitive practice.	langweilte mich der gleichbleibende Ablauf.

Note. Answer format for the English items ranges from 1 (*completely disagree*) to 5 (*completely agree*). Answer format for the German items ranges from 1 (*stimmt gar nicht*) to 5 (*stimmt genau*).

SM1_5: ***Silence Retreat Boredom Scale – Trait (SRBS-Trait)***

Nr.	English	German
1	During silence retreats, I usually get bored.	Schweigeretreats langweilen mich üblicherweise.
2	I usually get bored in silence at silence retreats.	Beim Schweigen bei Schweigeretreats langweile ich mich üblicherweise.
3	The long, silent sitting during silence retreats usually bores me.	Das lange, stille Sitzen bei Schweigeretreats langweilt mich üblicherweise.
4	I usually get bored with the physical exercises during silence retreats.	Bei den körperlichen Übungen bei Schweigeretreats langweile ich mich üblicherweise.
5	The meditation at silence retreats usually bores me.	Die Meditation bei Schweigeretreats langweilt mich üblicherweise.
6	The breathing exercises at silence retreats usually bore me.	Die Atemübungen bei Schweigeretreats langweilen mich üblicherweise.
7	The lectures on spiritual topics at silence retreats usually bore me.	Die Vorträge über spirituelle Themen bei Schweigeretreats langweilen mich üblicherweise.
8	The recitation of mantras or prayers at silence retreats usually bores me.	Das Rezitieren von Mantren oder Gebeten bei Schweigeretreats langweilt mich üblicherweise.
9	I am usually bored in the community at silence retreats.	In der Gemeinschaft bei Schweigeretreats langweile ich mich üblicherweise.
10	The leader of the silence retreat usually bores me.	Der Leiter, die Leiterin des Schweigeretreats langweilt mich üblicherweise.
11	I usually get bored of the same routine at silence retreats.	Der gleichbleibende Ablauf bei Schweigeretreats langweilt mich üblicherweise.

Note. Answer format for the English items ranges from 1 (*completely disagree*) to 5 (*completely agree*). Answer format for the German items ranges from 1 (*stimmt gar nicht*) to 5 (*stimmt genau*).

SM1_6: *Silence Retreat Boredom Scale – State (SRBS-State)*

Nr.	English	German
	In the current or completed silence retreat …	Bei dem aktuellen, oder abgeschlossenem Schweigeretreat …
1	I was bored.	langweilte ich mich.
2	I was bored with the silence.	langweilte mich das Schweigen.
3	I was bored of sitting in silence for a long time.	langweilte mich das lange, stille Sitzen.
4	I was bored with the physical exercises.	langweilten mich die körperlichen Übungen.
5	I was bored with the meditations.	langweilten mich die Meditationen.
6	I was bored with the breathing exercises.	langweilten mich die Atemübungen.
7	I was bored with the lectures on spiritual topics.	langweilten mich die Vorträge zu spirituellen Themen.
8	I was bored with the recitation of mantras or prayers.	langweilte mich das Rezitieren von Mantren oder Gebeten.
9	I was bored with the community.	langweilte mich die Gemeinschaft.
10	I was bored of the retreat leader.	langweilte mich die Leitung des Retreats.
11	I was bored by the repetitive schedule.	langweilte mich der gleichbleibende Ablauf.

Note. Answer format for the English items ranges from 1 (*completely disagree*) to 5 (*completely agree*). Answer format for the German items ranges from 1 (*stimmt gar nicht*) to 5 (*stimmt genau*).

SM1_7: *Sermon Boredom Scale – Trait (SBS-T)*

Nr.	English	German
1	I could fall asleep during the sermon.	Während der Predigt könnte ich einschlafen.
2	I generally find sermons boring.	Ich empfinde Predigten allgemein als langweilig.
3	My thoughts often wander during the sermon, so that my mind is somewhere else entirely.	Während der Predigt schweifen meine Gedanken oft umher, sodass ich gedanklich ganz woanders bin.
4	During the sermon, I have the feeling that time passes more slowly than usual.	Während der Predigt habe ich das Gefühl, dass die Zeit langsamer vergeht als sonst.
5	I am easily distracted during the sermon.	Während der Predigt bin ich leicht abzulenken.
6	I find that listening to the sermon doesn’t do much for me.	Ich finde. dass mir das Hören der Predigt nicht viel bringt.
7	I find the content of the sermon uninteresting.	Den Inhalt der Predigt finde ich uninteressant.

Note. Answer format for the English items ranges from 1 (*completely disagree*) to 5 (*completely agree*). Answer format for the German items ranges from 1 (*stimmt gar nicht*) to 5 (*stimmt genau*).

SM1_8: ***Sermon Boredom Scale – State (SBS-S)***

Nr.	English	German
1	I could have fallen asleep during the sermon.	Während der Predigt hätte ich einschlafen können.
2	I found the sermon boring today.	Ich fand die Predigt heute langweilig.
3	My thoughts often wandered during the sermon so that my mind was somewhere else.	Während der Predigt sind meine Gedanken oft umher geschweift, sodass ich gedanklich ganz woanders war.
4	During the sermon, I had the feeling that time passed more slowly than usual.	Während der Predigt hatte ich das Gefühl, dass die Zeit langsamer vergeht als sonst.
5	I was easily distracted during the sermon.	Während der Predigt war ich leicht abzulenken.
6	I don't think I gained much from listening to the sermon.	Ich finde, dass mir das Hören der Predigt nicht viel gebracht hat.
7	I found the content of today's sermon uninteresting.	Den Inhalt der heutigen Predigt fand ich uninteressant.

Note. Answer format for the English items ranges from 1 (*completely disagree*) to 5 (*completely agree*). Answer format for the German items ranges from 1 (*stimmt gar nicht*) to 5 (*stimmt genau*).

SM1_9: ***Pilgrimage Boredom Scale – Trait (PBS-Trait)***

Nr.	English	German
1	I usually get bored when I'm on a pilgrimage.	Beim Pilgern langweile ich mich üblicherweise.
2	Walking on a pilgrimage usually bores me.	Das Wandern beim Pilgern langweilt mich üblicherweise.
3	I am usually bored by the encounters I have on a pilgrimage.	Die Begegnungen beim Pilgern langweilen mich üblicherweise.
4	I am usually bored by nature on a pilgrimage.	Die Natur beim Pilgern langweilt mich üblicherweise.
5	I am usually bored by the peace and quiet of pilgrimage.	Die Ruhe beim Pilgern langweilt mich üblicherweise.
6	The distance from everyday life usually bores me.	Der Abstand vom Alltag langweilt mich üblicherweise.
7	I am usually bored by praying and church services.	Das Beten und die Gottesdienste langweilen mich üblicherweise.
8	The spiritual places usually bore me.	Die spirituellen Orte langweilen mich üblicherweise.
9	I am usually bored by the self-experiences of pilgrimage.	Die Selbsterfahrungen des Pilgerns langweilen ich mich üblicherweise.
10	The search for myself usually bores me.	Die Suche nach mir Selbst langweilt mich üblicherweise.
11	I usually get bored of the same routine on a pilgrimage.	Der gleichbleibende Ablauf des Pilgerns langweilt mich üblicherweise.

Note. Answer format for the English items ranges from 1 (*completely disagree*) to 5 (*completely agree*). Answer format for the German items ranges from 1 (*stimmt gar nicht*) to 5 (*stimmt genau*).

SM1_10: ***Pilgrimage Boredom Scale – State (PBS-State)***

Nr.	English	German
	In the last few days of my pilgrimage ...	In den letzten Tagen meiner Pilgerreise ...
1	I was bored.	langweilte ich mich.
2	I was bored of walking.	langweilte mich das Wandern.
3	I was bored with the encounters on the pilgrimage.	langweilten mich die Begegnungen beim Pilgern.
4	I was bored with nature.	langweilte mich die Natur.
5	I was bored with the peace and quiet.	langweilte mich die Ruhe.
6	I was bored of the distance from everyday life.	langweilte mich der Abstand vom Alltag.
7	I was bored of praying and church services.	langweilten mich das Beten und die Gottesdienste.
8	I was bored of spiritual places.	langweilten mich die spirituellen Orte.
9	I was bored with the self-awareness of pilgrimage.	langweilten mich die Selbsterfahrungen des Pilgerns.
10	I was bored with the search for myself.	langweilte mich die Suche nach mir Selbst.
11	I was bored with the repetitive nature of the pilgrimage.	langweilte mich der gleichbleibende Ablauf des Pilgerns.

Note. Answer format for the English items ranges from 1 (*completely disagree*) to 5 (*completely agree*). Answer format for the German items ranges from 1 (*stimmt gar nicht*) to 5 (*stimmt genau*).

SM1_11: ***Multidimensional State Boredom Scale (MSBS)***

Nr.	English	German
1	I felt like things I did had no value to me.	Ich habe das Gefühl, Dinge, die ich gemacht habe, hatten keinen Wert für mich.
2	I felt bored.	Ich fühlte mich gelangweilt.
3	I was wasting time that could be better spent elsewhere.	Ich verschwendete Zeit, die woanders besser verwendet werden konnte.
4	I wanted something to happen, but I didn't know what.	Ich wollte, dass etwas passiert, aber ich wusste nicht was.
5	I felt like I was sitting around waiting for something.	Ich hatte das Gefühl herumzusitzen und auf etwas zu warten.
6	I was easily distracted.	Ich war leicht abgelenkt.
7	My mind was wandering.	Meine Gedanken wanderten herum.
8	Time passed more slowly than usual.	Die Zeit verging langsamer als gewöhnlich.

Note. Answer format for the English items ranges from 1 (*completely disagree*) to 5 (*completely agree*). Answer format for the German items ranges from 1 (*stimmt gar nicht*) to 5 (*stimmt genau*).

SM2: Assessments of Over- and Underchallenge

The German version of the items was used in this study. The English version has been translated via artificial intelligence (deepl.com) and has not yet been psychometrically evaluated.

SM2_1: ***Overchallenge Assessment – Study 1 (Yoga-Trait)***

Nr.	English	German
1	The yoga practice usually overchallenges me.	Die Yoga-Praxis überfordert mich üblicherweise.
2	The physical exercises usually overchallenge me.	Die Körperübungen überfordern mich üblicherweise.
3	The breathing exercises usually overchallenge me.	Die Atemübungen überfordern mich üblicherweise.
4	The meditation usually overchallenges me.	Die Meditation überfordert mich üblicherweise.
5	The relaxation phase usually overchallenges me.	Die Entspannungsphase überfordert mich üblicherweise.

Note. Answer format for the English items ranges from 1 (*completely disagree*) to 5 (*completely agree*). Answer format for the German items ranges from 1 (*stimmt gar nicht*) to 5 (*stimmt genau*).

SM2_2: ***Underchallenge Assessment – Study 1 (Yoga-Trait)***

Nr.	English	German
1	The yoga practice usually underchallenges me.	Die Yoga-Praxis unterfordert mich üblicherweise.
2	The physical exercises usually underchallenge me.	Die Körperübungen unterfordert mich üblicherweise.
3	The breathing exercises usually underchallenge me.	Die Atemübungen unterfordern mich üblicherweise.
4	The meditation is usually too challenging for me.	Die Meditation unterfordert mich üblicherweise.
5	The relaxation phase is usually not challenging enough for me.	Die Entspannungsphase unterfordert mich üblicherweise.

Note. Answer format for the English items ranges from 1 (*completely disagree*) to 5 (*completely agree*). Answer format for the German items ranges from 1 (*stimmt gar nicht*) to 5 (*stimmt genau*).

SM2_3: ***Overchallenge Assessment – Study 2 (Yoga-State)***

**Nr.**	**English**	**German**
1	I felt overchallenged during the yoga session I just completed.	In der gerade absolvierten Yoga-Einheit fühlte ich mich überfordert.
2	The physical exercises overchallenged me.	Die Körperübungen überforderten mich.
3	The breathing exercises overchallenged me.	Die Atemübungen überforderten mich.
4	The meditation overchallenged me	Die Meditation überforderte mich
5	The relaxation phase overchallenged me.	Die Entspannungsphase überforderte mich.

Note. Answer format for the English items ranges from 1 (*completely disagree*) to 5 (*completely agree*). Answer format for the German items ranges from 1 (*stimmt gar nicht*) to 5 (*stimmt genau*).

SM2_4: ***Underchallenge Assessment – Study 2 (Yoga-State)***

Nr.	English	German
1	I felt underchallenged during the yoga session I just completed.	In der gerade absolvierten Yoga-Einheit fühlte ich mich unterfordert.
2	The physical exercises underchallenged me.	Die Körperübungen unterforderten mich.
3	The breathing exercises underchallenged me.	Die Atemübungen unterforderten mich.
4	The meditation underchallenged me	Die Meditation unterforderte mich.
5	The relaxation phase underchallenged me.	Die Entspannungsphase unterforderte mich.

Note. Answer format for the English items ranges from 1 (*completely disagree*) to 5 (*completely agree*). Answer format for the German items ranges from 1 (*stimmt gar nicht*) to 5 (*stimmt genau*).

SM2_5: ***Overchallenge Assessment – Study 3 (Meditation-Trait)***

Nr.	English	German
1	Meditation usually overchallenges me.	Die Meditation überfordert mich üblicherweise.
2	The concentration exercises usually overchallenge me.	Die Konzentrationsübungen überfordern mich üblicherweise.
3	The breathing exercises usually overchallenge me.	Die Atemübungen überfordern mich üblicherweise.
4	The relaxation phase usually overchallenge me.	Die Entspannungsphase überfordert mich üblicherweise.

Note. Answer format for the English items ranges from 1 (*completely disagree*) to 5 (*completely agree*). Answer format for the German items ranges from 1 (*stimmt gar nicht*) to 5 (*stimmt genau*).

SM2_6: ***Underchallenge Assessment – Study 3 (Meditation-Trait)***

Nr.	English	German
1	Meditation usually underchallenges me.	Die Meditation unterfordert mich üblicherweise.
2	The concentration exercises usually underchallenge me.	Die Konzentrationsübungen unterfordern mich üblicherweise.
3	The breathing exercises usually underchallenge me.	Die Atemübungen unterfordern mich üblicherweise.
4	The relaxation phase is usually not challenging enough for me.	Die Entspannungsphase unterfordert mich üblicherweise.

Note. Answer format for the English items ranges from 1 (*completely disagree*) to 5 (*completely agree*). Answer format for the German items ranges from 1 (*stimmt gar nicht*) to 5 (*stimmt genau*).

SM2_7: ***Overchallenge Assessment – Study 4 (Meditation-State)***

Nr.	English	German
1	I felt overchallenged during the meditation session I just completed.	In der gerade absolvierten Meditationseinheit fühlte ich mich überfordert.
2	The physical exercises overchallenged me.	Die Konzentrationsübungen überforderten mich.
3	The breathing exercises overchallenged me.	Die Atemübungen überforderten mich.
4	The relaxation phase overchallenged me.	Die Entspannungsphase überforderte mich.

Note. Answer format for the English items ranges from 1 (*completely disagree*) to 5 (*completely agree*). Answer format for the German items ranges from 1 (*stimmt gar nicht*) to 5 (*stimmt genau*).

SM2_8: ***Underchallenge Assessment – Study 4 (Meditation-State)***

Nr.	English	German
1	I felt underchallenged during the meditation session I just completed.	In der gerade absolvierten Meditationseinheit fühlte ich mich unterfordert.
2	The physical exercises underchallenged me.	Die Konzentrationsübungen unterforderten mich.
3	The breathing exercises underchallenged me.	Die Atemübungen unterforderten mich.
4	The relaxation phase underchallenged me.	Die Entspannungsphase unterforderte mich.

Note. Answer format for the English items ranges from 1 (*completely disagree*) to 5 (*completely agree*). Answer format for the German items ranges from 1 (*stimmt gar nicht*) to 5 (*stimmt genau*).

SM2_9: ***Overchallenge Assessment – Study 5 (Silence Retreat-Trait)***

Nr.	English	German
1	Silence retreats usually overchallenge me.	Schweigeretreats überfordern mich üblicherweise.
2	Maintaining silence and permanent stillness usually overchallenge me.	Das Schweigen und die dauerhafte Stille einzuhalten, überfordern mich üblicherweise.
3	Keeping to a strict schedule is usually too much for me.	Den strikten Zeitplan einzuhalten überfordert mich üblicherweise.
4	Sitting in one position for a long time usually overchallenges me.	Das lange Sitzen in einer Position überfordert mich üblicherweise.
5	The physical exercises usually overchallenge me.	Die körperlichen Übungen überfordern mich üblicherweise.
6	The meditation techniques usually overchallenge me.	Die Meditationstechniken überfordern mich üblicherweise.
7	The breathing exercises usually overchallenge me.	Die Atemübungen überfordern mich üblicherweise.
8	Lectures on spiritual topics usually overchallenge me.	Die Vorträge zu spirituellen Themen überfordern mich üblicherweise.
9	The recitation of mantras or prayers at silent retreats usually overchallenges me.	Das Rezitieren von Mantren oder Gebeten bei Schweigeretreats überfordert mich üblicherweise.
10	The community at silent retreats usually overchallenges me.	Die Gemeinschaft bei Schweigeretreats überfordert mich üblicherweise.

Note. Answer format for the English items ranges from 1 (*completely disagree*) to 5 (*completely agree*). Answer format for the German items ranges from 1 (*stimmt gar nicht*) to 5 (*stimmt genau*).

SM2_10: ***Underchallenge Assessment – Study 5 (Silence Retreat-Trait)***

Nr.	English	German
1	Silence retreats usually underchallenge me.	Schweigeretreats unterfordern mich üblicherweise.
2	Maintaining silence and permanent stillness usually underchallenge me.	Das Schweigen und die dauerhafte Stille einzuhalten, unterfordern mich üblicherweise.
3	Keeping to a strict schedule is usually underchallenging for me.	Den strikten Zeitplan einzuhalten unterfordert mich üblicherweise.
4	Sitting in one position for a long time usually underchallenges me.	Das lange Sitzen in einer Position unterfordert mich üblicherweise.
5	The physical exercises usually underchallenge me.	Die körperlichen Übungen unterfordern mich üblicherweise.
6	The meditation techniques usually underchallenge me.	Die Meditationstechniken unterfordern mich üblicherweise.
7	The breathing exercises usually underchallenge me.	Die Atemübungen unterfordern mich üblicherweise.
8	Lectures on spiritual topics usually underchallenge me.	Die Vorträge über spirituelle Themen unterfordern mich üblicherweise.
9	The recitation of mantras or prayers at silent retreats usually underchallenges me.	Das Rezitieren von Mantren oder Gebeten bei Schweigeretreats unterfordert mich üblicherweise.
10	The community at silent retreats usually underchallenges me.	Die Gemeinschaft bei Schweigeretreats unterfordert mich üblicherweise.

Note. Answer format for the English items ranges from 1 (*completely disagree*) to 5 (*completely agree*). Answer format for the German items ranges from 1 (*stimmt gar nicht*) to 5 (*stimmt genau*).

SM2_11: ***Overchallenge Assessment – Study 6 (Silence Retreat-State)***

Nr.	English	German
1	Silence retreats overchallenge me.	Schweigeretreats überfordern mich.
2	Maintaining silence and permanent stillness is too much for me.	Das Schweigen und die dauerhafte Stille einzuhalten, überfordern mich.
3	Keeping to a strict schedule is too much for me.	Den strikten Zeitplan einzuhalten überfordert mich.
4	Sitting in one position for a long time is too much for me.	Das lange Sitzen in einer Position überfordert mich.
5	The physical exercises overchallenge me.	Die körperlichen Übungen überfordern mich.
6	The meditation techniques overchallenge me.	Die Meditationstechniken überfordern mich.
7	The breathing exercises overchallenge me.	Die Atemübungen überfordern mich.
8	Lectures on spiritual topics overchallenge me.	Die Vorträge über spirituelle Themen überfordern mich.
9	The recitation of mantras or prayers at silence retreats overchallenges me.	Das Rezitieren von Mantren oder Gebeten bei Schweigeretreats überfordert mich.
10	The community at silence retreats overchallenges me.	Die Gemeinschaft bei Schweigeretreats überfordert mich.

Note. Answer format for the English items ranges from 1 (*completely disagree*) to 5 (*completely agree*). Answer format for the German items ranges from 1 (*stimmt gar nicht*) to 5 (*stimmt genau*).

SM2_12: ***Underchallenge Assessment – Study 6 (Silence Retreat-State)***

Nr.	English	German
1	Silence retreats underchallenge me.	Schweigeretreats unterfordern mich.
2	Maintaining silence and permanent stillness is too much for me.	Das Schweigen und die dauerhafte Stille einzuhalten, unterfordern mich.
3	Keeping to a strict schedule is too much for me.	Den strikten Zeitplan einzuhalten unterfordert mich.
4	Sitting in one position for a long time is too much for me.	Das lange Sitzen in einer Position unterfordert mich.
5	The physical exercises underchallenge me.	Die körperlichen Übungen unterfordern mich.
6	The meditation techniques underchallenge me.	Die Meditationstechniken unterfordern mich.
7	The breathing exercises underchallenge me.	Die Atemübungen unterfordern mich.
8	Lectures on spiritual topics underchallenge me.	Die Vorträge über spirituelle Themen unterfordern mich.
9	The recitation of mantras or prayers at silence retreats underchallenges me.	Das Rezitieren von Mantren oder Gebeten bei Schweigeretreats unterfordert mich.
10	The community at silence retreats underchallenges me.	Die Gemeinschaft bei Schweigeretreats unterfordert mich.

Note. Answer format for the English items ranges from 1 (*completely disagree*) to 5 (*completely agree*). Answer format for the German items ranges from 1 (*stimmt gar nicht*) to 5 (*stimmt genau*).

SM2_13: ***Overchallenge Assessment – Study 7 (Sermon-Trait)***

Nr.	English	German
1	The sermon overchallenges me.	Die Predigt überfordert mich.

Note. Answer format for the English items ranges from 1 (*completely disagree*) to 5 (*completely agree*). Answer format for the German items ranges from 1 (*stimmt gar nicht*) to 5 (*stimmt genau*).

SM2_14: ***Underchallenge Assessment – Study 7 (Sermon-Trait)***

Nr.	English	German
1	The sermon underchallenges me.	Die Predigt unterfordert mich.

Note. Answer format for the English items ranges from 1 (*completely disagree*) to 5 (*completely agree*). Answer format for the German items ranges from 1 (*stimmt gar nicht*) to 5 (*stimmt genau*).

SM2_15: *Overchallenge Assessment – Study 8 (Sermon-State)*

Nr.	English	German
1	The content of the sermon overchallenged me today.	Der Inhalt der Predigt hat mich heute überfordert.

Note. Answer format for the English items ranges from 1 (*completely disagree*) to 5 (*completely agree*). Answer format for the German items ranges from 1 (*stimmt gar nicht*) to 5 (*stimmt genau*).

SM2_16: ***Assessment – Study 8 (Sermon-State)***

Nr.	English	German
1	The content of the sermon underchallenged me today.	Der Inhalt der Predigt hat mich heute unterfordert.

Note. Answer format for the English items ranges from 1 (*completely disagree*) to 5 (*completely agree*). Answer format for the German items ranges from 1 (*stimmt gar nicht*) to 5 (*stimmt genau*).

SM2_17: ***Overchallenge Assessment – Study 9 (Pilgrimage-Trait)***

Nr.	English	German
1	Pilgrimages usually overchallenge me.	Das Pilgern überfordert mich üblicherweise.
2	Walking on a pilgrimage usually overchallenges me.	Das Wandern beim Pilgern überfordert mich üblicherweise.
3	The encounters on a pilgrimage usually overchallenge me.	Die Begegnungen beim Pilgern überfordern mich üblicherweise.
4	The peace and quiet usually overchallenges me.	Die Ruhe überfordert mich üblicherweise.
5	The distance from everyday life usually overchallenges me.	Der Abstand vom Alltag überfordert mich üblicherweise.
6	Praying and church services usually overchallenge me.	Das Beten und die Gottesdienste überfordern mich üblicherweise.
7	The spiritual places usually overchallenge me.	Die spirituellen Orte überfordern mich üblicherweise.
8	The self-awareness of pilgrimage usually overchallenges me.	Die Selbsterfahrungen des Pilgerns überfordern mich üblicherweise.
9	The search for myself usually overchallenges me.	Die Suche nach mir Selbst überfordert mich üblicherweise.
10	The constant sequence of the pilgrimage usually overchallenges me.	Der gleichbleibende Ablauf des Pilgerns überfordert mich üblicherweise.
11	The weather conditions usually overchallenge me.	Die Wetterbedingungen überfordern mich üblicherweise.
12	The sleeping conditions usually overchallenge me.	Die Schlafbedingungen überfordern mich üblicherweise.

Note. Answer format for the English items ranges from 1 (*completely disagree*) to 5 (*completely agree*). Answer format for the German items ranges from 1 (*stimmt gar nicht*) to 5 (*stimmt genau*).

SM2_18: ***Underchallenge Assessment – Study 9 (Pilgrimage-Trait)***

Nr.	English	German
1	Pilgrimages usually underchallenge me.	Das Pilgern unterfordert mich üblicherweise.
2	Walking on a pilgrimage usually underchallenges me.	Das Wandern beim Pilgern unterfordert mich üblicherweise.
3	The encounters on a pilgrimage usually underchallenge me.	Die Begegnungen beim Pilgern unterfordern mich üblicherweise.
4	The peace and quiet usually underchallenges me.	Die Ruhe unterfordert mich üblicherweise.
5	The distance from everyday life usually underchallenges me.	Der Abstand vom Alltag unterfordert mich üblicherweise.
6	Praying and church services usually underchallenge me.	Das Beten und die Gottesdienste unterfordern mich üblicherweise.
7	The spiritual places usually underchallenge me.	Die spirituellen Orte unterfordern mich üblicherweise.
8	The self-awareness of pilgrimage usually underchallenges me.	Die Selbsterfahrungen des Pilgerns unterfordern mich üblicherweise.
9	The search for myself usually underchallenges me.	Die Suche nach mir Selbst unterfordert mich üblicherweise.
10	The constant sequence of the pilgrimage usually underchallenges me.	Der gleichbleibende Ablauf des Pilgerns unterfordert mich üblicherweise.
11	The weather conditions usually underchallenge me.	Mit den Wetterbedingungen habe ich üblicherweise keine Probleme.
12	The sleeping conditions usually underchallenge me.	Mit den Schlafbedingungen komme ich üblicherweise gut zurecht.

Note. Answer format for the English items ranges from 1 (*completely disagree*) to 5 (*completely agree*). Answer format for the German items ranges from 1 (*stimmt gar nicht*) to 5 (*stimmt genau*).

SM2_19: ***Overchallenge Assessment – Study 10 (Pilgrimage-State)***

Nr.	English	German
1	The pilgrimage overchallenged me.	Das Pilgern überforderte mich.
2	Walking on a pilgrimage was overchallenging for me.	Das Wandern beim Pilgern überforderte mich.
3	The encounters on the pilgrimage overchallenged me.	Die Begegnungen beim Pilgern überforderten mich.
4	The peace and quiet overchallenged me.	Die Ruhe überforderte mich.
5	The distance from everyday life overchallenged me.	Der Abstand vom Alltag überforderte mich.
6	The praying and the church services overchallenged me.	Das Beten und die Gottesdienste überforderten mich.
7	The spiritual places overchallenged me.	Die spirituellen Orte überforderten mich.
8	The self-awareness of the pilgrimage overchallenged me.	Die Selbsterfahrungen des Pilgerns überforderten mich.
9	The search for myself overchallenged me.	Die Suche nach mir Selbst überforderte mich.
10	The unchanging course of the pilgrimage overchallenged me.	Der gleichbleibende Ablauf des Pilgerns überforderte mich.
11	The weather conditions overchallenged me.	Die Wetterbedingungen überforderten mich.
12	The sleeping conditions overchallenged me.	Die Schlafbedingungen überforderten mich.

Note. Answer format for the English items ranges from 1 (*completely disagree*) to 5 (*completely agree*). Answer format for the German items ranges from 1 (*stimmt gar nicht*) to 5 (*stimmt genau*).

SM2_20: ***Underchallenge Assessment – Study 10 (Pilgrimage-State)***

Nr.	English	German
1	The pilgrimage underchallenged me.	Das Pilgern unterforderte mich.
2	Walking on a pilgrimage was underchallenging for me.	Das Wandern beim Pilgern unterforderte mich.
3	The encounters on the pilgrimage underchallenged me.	Die Begegnungen beim Pilgern unterforderten mich.
4	The peace and quiet underchallenged me.	Die Ruhe unterforderte mich.
5	The distance from everyday life underchallenged me.	Der Abstand vom Alltag unterforderte mich.
6	The praying and the church services underchallenged me.	Das Beten und die Gottesdienste unterforderten mich.
7	The spiritual places underchallenged me.	Die spirituellen Orte unterforderte mich.
8	The self-awareness of the pilgrimage underchallenged me.	Die Selbsterfahrungen des Pilgerns unterforderten mich.
9	The search for myself underchallenged me.	Die Suche nach mir Selbst unterforderte mich.
10	The unchanging course of the pilgrimage underchallenged me.	Der gleichbleibende Ablauf des Pilgerns unterforderte mich.
11	The weather conditions underchallenged me.	Mit den Wetterbedingungen hatte ich keine Probleme.
12	The sleeping conditions underchallenged me.	Mit den Schlafbedingungen kam ich gut zurecht.

Note. Answer format for the English items ranges from 1 (*completely disagree*) to 5 (*completely agree*). Answer format for the German items ranges from 1 (*stimmt gar nicht*) to 5 (*stimmt genau*).

SM3: Assessments of Value
The German version of the items was used in this study. The English version has been translated via artificial intelligence (deepl.com) and has not yet been psychometrically evaluated.

SM3_1: ***Value Assessment – Study 1 (Yoga-Trait)***

Nr.	English	German
1	The physical exercises in yoga are important to me.	Die Körperübungen beim Yoga sind mir wichtig.
2	The breathing exercises in yoga are important to me.	Die Atemübungen beim Yoga sind mir wichtig.
3	Meditation in yoga is important to me.	Die Meditation beim Yoga ist mir wichtig.
4	Relaxation in yoga is important to me.	Die Entspannung beim Yoga ist mir wichtig.
5	The group meetings and social interaction in yoga are important to me.	Die Gruppentreffen und der soziale Austausch beim Yoga sind mir wichtig.
6	The spiritual aspects of yoga are important to me.	Die spirituellen Aspekte an Yoga sind mir wichtig.
7	Yoga is important to me.	Yoga ist mir wichtig.
8	Yoga has a high priority in my life.	Yoga hat einen hohen Stellenwert in meinem Leben.
9	Regardless of how good others are at yoga, it is important to me to be good at yoga.	Ganz unabhängig davon, wie gut andere im Yoga sind, ist es mir wichtig, gut im Yoga zu sein.
10	It is important to me to be good at yoga.	Es ist mir wichtig gut in Yoga zu sein.
11	It is important to me to be better at yoga than others.	Es ist mir wichtig, besser als die anderen in Yoga zu sein.

Note. Answer format for the English items ranges from 1 (*completely disagree*) to 5 (*completely agree*). Answer format for the German items ranges from 1 (*stimmt gar nicht*) to 5 (*stimmt genau*).

SM3_2: ***Value Assessment – Study 2 (Yoga-State)***

Nr.	English	German
1	The physical exercises in yoga are important to me.	Die Körperübungen beim Yoga sind mir wichtig.
2	The breathing exercises in yoga are important to me.	Die Atemübungen beim Yoga sind mir wichtig.
3	Meditation in yoga is important to me.	Die Meditation beim Yoga ist mir wichtig.
4	Relaxation in yoga is important to me.	Die Entspannung beim Yoga ist mir wichtig.
5	The group meetings and social interaction in yoga are important to me.	Die Gruppentreffen und der soziale Austausch beim Yoga sind mir wichtig.
6	The spiritual aspects of yoga are important to me.	Die spirituellen Aspekte an Yoga sind mir wichtig.
7	Yoga is important to me.	Yoga ist mir wichtig.
8	Yoga has a high priority in my life.	Yoga hat einen hohen Stellenwert in meinem Leben.
9	Regardless of how good others are at yoga, it is important to me to be good at yoga.	Ganz unabhängig davon, wie gut andere im Yoga sind, ist es mir wichtig, gut im Yoga zu sein.
10	It is important to me to be good at yoga.	Es ist mir wichtig gut in Yoga zu sein.
11	It is important to me to be better at yoga than others.	Es ist mir wichtig, besser als die anderen in Yoga zu sein.

Note. Answer format for the English items ranges from 1 (*completely disagree*) to 5 (*completely agree*). Answer format for the German items ranges from 1 (*stimmt gar nicht*) to 5 (*stimmt genau*).

SM3_3: ***Value Assessment – Study 3 (Meditation-Trait)***

Nr.	English	German
1	The breathing exercises during meditation are important to me.	Die Atemübungen bei der Meditation sind mir wichtig.
2	The concentration exercises during meditation are important to me.	Die Konzentrationsübungen bei der Meditation sind mir wichtig.
3	Relaxation during meditation is important to me.	Die Entspannung bei der Meditation ist mir wichtig.
4	Relaxation during yoga is important to me.	Die Entspannung beim Yoga ist mir wichtig.
5	The group meetings and social interaction during meditation are important to me.	Die Gruppentreffen und der soziale Austausch bei der Meditation sind mir wichtig.
6	The spiritual aspects of meditation are important to me.	Die spirituellen Aspekte an Meditation sind mir wichtig.
7	Meditation is important to me.	Meditation ist mir wichtig.
8	Meditation has a high priority in my life.	Meditation hat einen hohen Stellenwert in meinem Leben.
9	Regardless of how good others are at meditation, it is important to me to be good at meditation.	Ganz unabhängig davon, wie gut andere im Meditieren sind, ist es mir wichtig, gut im Meditieren zu sein.
10	It is important to me to be good at yoga.	Es ist mir wichtig gut in Yoga zu sein.
11	It is important to me to be better at meditation than others.	Es ist mir wichtig, besser als die anderen in Meditation zu sein.

Note. Answer format for the English items ranges from 1 (*completely disagree*) to 5 (*completely agree*). Answer format for the German items ranges from 1 (*stimmt gar nicht*) to 5 (*stimmt genau*).

SM3_4: ***Value Assessment – Study 4 (Meditation-State)***

Nr.	English	German
1	The breathing exercises during meditation are important to me.	Die Atemübungen bei der Meditation sind mir wichtig.
2	The concentration exercises during meditation are important to me.	Die Konzentrationsübungen bei der Meditation sind mir wichtig.
3	Relaxation during meditation is important to me.	Die Entspannung bei der Meditation ist mir wichtig.
4	The group meetings and social interaction during meditation are important to me.	Die Gruppentreffen und der soziale Austausch bei der Meditation sind mir wichtig.
5	The spiritual aspects of meditation are important to me.	Die spirituellen Aspekte an Meditation sind mir wichtig.
6	Meditation is important to me.	Meditation ist mir wichtig.
7	Meditation has a high priority in my life.	Meditation hat einen hohen Stellenwert in meinem Leben.
8	Regardless of how good others are at meditating, it is important to me to be good at meditating.	Ganz unabhängig davon, wie gut andere im Meditieren sind, ist es mir wichtig, gut im Meditieren zu sein.
9	It is important to me to be better at meditation than others.	Es ist mir wichtig, besser als die anderen in Meditation zu sein.

Note. Answer format for the English items ranges from 1 (*completely disagree*) to 5 (*completely agree*). Answer format for the German items ranges from 1 (*stimmt gar nicht*) to 5 (*stimmt genau*).

SM3_5: ***Value Assessment – Study 5 (Silence Retreat-Trait)***

Nr.	English	German
1	Silence during silent retreats is important to me.	Das Schweigen bei Schweigeretreats ist mir wichtig.
2	The long, silent sitting during silent retreats is important to me.	Das lange, stille Sitzen bei Schweigeretreats ist mir wichtig.
3	The physical exercises during silent retreats are important to me.	Die körperlichen Übungen bei Schweigeretreats sind mir wichtig.
4	The meditation during silent retreats is important to me.	Die Meditation bei Schweigeretreats ist mir wichtig.
5	The breathing exercises at silent retreats are important to me.	Die Atemübungen bei Schweigeretreats sind mit wichtig.
6	The talks on spiritual topics at silent retreats are important to me.	Die Vorträge über spirituelle Themen bei Schweigeretreats sind mir wichtig.
7	The recitation of mantras or prayers at silent retreats is important to me.	Das Rezitieren von Mantren oder Gebeten bei Schweigeretreats ist mir wichtig.
8	The community at silent retreats is important to me.	Die Gemeinschaft bei Schweigeretreats ist mir wichtig.
9	The leader of the silent retreat is important to me.	Der Leiter, die Leiterin des Schweigeretreats ist mir wichtig.
10	The consistent schedule at silent retreats is important to me.	Der gleichbleibende Ablauf bei Schweigeretreats ist mir wichtig.
11	Silent retreats are important to me.	Schweigeretreats sind mir wichtig.
12	Silent retreats have a high priority in my life.	Schweigeretreats haben einen hohen Stellenwert in meinem Leben.
13	It is important to me to be good at the activities of the silent retreats.	Es ist mir wichtig, gut bei den Aktivitäten der Schweigeretreats zu sein.

Note. Answer format for the English items ranges from 1 (*completely disagree*) to 5 (*completely agree*). Answer format for the German items ranges from 1 (*stimmt gar nicht*) to 5 (*stimmt genau*).

SM3_6: ***Value Assessment – Study 6 (Silence Retreat-State)***

Nr.	English	German
1	The silence during the silence retreat was important to me.	Das Schweigen beim Schweigeretreat war mir wichtig.
2	The long, silent sitting during the silence retreat was important to me.	Das lange, stille Sitzen beim Schweigeretreat war mir wichtig.
3	The physical exercises during the silence retreat were important to me.	Die körperlichen Übungen beim Schweigeretreat waren mir wichtig.
4	The meditation during the silence retreat was important to me.	Die Meditation beim Schweigeretreat war mir wichtig.
5	The breathing exercises at the silence retreat were important to me.	Die Atemübungen beim Schweigeretreat waren mir wichtig.
6	The talks on spiritual topics at the silence retreat were important to me.	Die Vorträge über spirituelle Themen beim Schweigeretreat waren mir wichtig.
7	The recitation of mantras or prayers at the silence retreat was important to me.	Das Rezitieren von Mantren oder Gebeten beim Schweigeretreat war mir wichtig.
8	The community at the silence retreat was important to me.	Die Gemeinschaft beim Schweigeretreat war mir wichtig.
9	The leader of the silence retreat was important to me.	Der Leiter, die Leiterin des Schweigeretreats war mir wichtig.
10	The consistent schedule of the silence retreat was important to me.	Der gleichbleibende Ablauf des Schweigeretreats war mir wichtig.
11	Silence retreats are important to me.	Schweigeretreats sind mir wichtig.
12	Silence retreats have a high priority in my life.	Schweigeretreats haben einen hohen Stellenwert in meinem Leben.
13	It was important to me to be good at the activities of the silence retreat.	Es war mir wichtig, gut bei den Aktivitäten des Schweigeretreats zu sein.

Note. Answer format for the English items ranges from 1 (*completely disagree*) to 5 (*completely agree*). Answer format for the German items ranges from 1 (*stimmt gar nicht*) to 5 (*stimmt genau*).

SM3_7: ***Value Assessment – Study 7 (Sermon-Trait)***

Nr.	English	German
1	Listening to the sermon fills my life with meaning.	Die Predigt zu hören, erfüllt mein Leben mit Sinn.
2	I find the content of the sermon useful for my life.	Den Inhalt der Predigt empfinde ich für mein Leben als nützlich.
3	To be honest, I don't care about the content of the sermon. (inverted)	Um ehrlich zu sein, ist mir der Inhalt der Predigt egal. (umgepolt)
4	Listening to the sermon is important to me.	Die Predigt zu hören ist mir wichtig.
5	The sermon is one of the highlights of the church service for me.	Die Predigt ist für mich einer der Höhepunkte des Gottesdienstes.
6	The sermon is very important in my life.	Die Predigt hat in meinem Leben einen hohen Stellenwert.
7	It is important to me to be perceived by other people as someone who is aware of the content of the sermon.	Mir ist es wichtig, von anderen Menschen als jemand wahrgenommen zu werden, der Kenntnis vom Inhalt der Predigt hat.

Note. Answer format for the English items ranges from 1 (*completely disagree*) to 5 (*completely agree*). Answer format for the German items ranges from 1 (*stimmt gar nicht*) to 5 (*stimmt genau*).

SM3_8: ***Value Assessment – Study 8 (Sermon-State)***

Nr.	English	German
1	Listening to the sermon fills my life with meaning.	Die Predigt zu hören, erfüllt mein Leben mit Sinn.
2	I find the content of the sermon useful for my life.	Den Inhalt der Predigt empfinde ich für mein Leben als nützlich.
3	To be honest, I usually don't care about the content of the sermon. (inverted)	Um ehrlich zu sein, ist mir der Inhalt der Predigt üblicherweise egal. (umgepolt)
4	Listening to the sermon is important to me.	Die Predigt zu hören ist mir wichtig.
5	The sermon is one of the highlights of the church service for me.	Die Predigt ist für mich einer der Höhepunkte des Gottesdienstes.
6	The sermon is very important in my life.	Die Predigt hat für mich einen hohen Stellenwert.
7	It is important to me to be perceived by other people as someone who is aware of the content of the sermon.	Mir ist es wichtig, von anderen Menschen als jemand wahrgenommen zu werden, der Kenntnis vom Inhalt der Predigt hat.

Note. Answer format for the English items ranges from 1 (*completely disagree*) to 5 (*completely agree*). Answer format for the German items ranges from 1 (*stimmt gar nicht*) to 5 (*stimmt genau*).

SM3_9: ***Value Assessment – Study 9 (Pilgrimage-Trait)***

Nr.	English	German
1	Walking on a pilgrimage is important to me.	Das Wandern beim Pilgern ist mir wichtig.
2	The encounters on a pilgrimage are important to me.	Die Begegnungen beim Pilgern sind mir wichtig.
3	Nature is important to me on a pilgrimage.	Die Natur beim Pilgern ist mir wichtig.
4	The peace and quiet on a pilgrimage is important to me.	Die Ruhe beim Pilgern ist mir wichtig.
5	The distance from everyday life is important to me.	Der Abstand vom Alltag ist mir wichtig.
6	Prayer and church services are important to me.	Das Beten und die Gottesdienste sind mir wichtig.
7	The spiritual places are important to me.	Die spirituellen Orte sind mir wichtig.
8	Experiencing myself on a pilgrimage is important to me.	Die Selbsterfahrungen beim Pilgern sind mir wichtig.
9	The search for myself is important to me.	Die Suche nach mir Selbst ist mir wichtig.
10	The consistent sequence of pilgrimages is important to me.	Der gleichbleibende Ablauf des Pilgerns ist mir wichtig.
11	Pilgrimage is important to me.	Pilgern ist mir wichtig.
12	Pilgrimage has a high priority in my life.	Pilgern hat einen hohen Stellenwert in meinem Leben.
13	It is important to me to be good at pilgrimage.	Es ist mir wichtig, gut im Pilgern zu sein.

Note. Answer format for the English items ranges from 1 (*completely disagree*) to 5 (*completely agree*). Answer format for the German items ranges from 1 (*stimmt gar nicht*) to 5 (*stimmt genau*).

SM3_10: ***Value Assessment – Study 10 (Pilgrimage-State)***

Nr.	English	German
1	Walking on a pilgrimage is important to me.	Das Wandern beim Pilgern ist mir wichtig.
2	The encounters on a pilgrimage are important to me.	Die Begegnungen beim Pilgern sind mir wichtig.
3	Nature is important to me on a pilgrimage.	Die Natur beim Pilgern ist mir wichtig.
4	The peace and quiet on a pilgrimage is important to me.	Die Ruhe beim Pilgern ist mir wichtig.
5	The distance from everyday life is important to me.	Der Abstand vom Alltag ist mir wichtig.
6	Prayer and church services are important to me.	Das Beten und die Gottesdienste sind mir wichtig.
7	The spiritual places are important to me.	Die spirituellen Orte sind mir wichtig.
8	Experiencing myself on a pilgrimage is important to me.	Die Selbsterfahrungen beim Pilgern sind mir wichtig.
9	The search for myself is important to me.	Die Suche nach mir Selbst ist mir wichtig.
10	The consistent sequence of pilgrimages is important to me.	Der gleichbleibende Ablauf des Pilgerns ist mir wichtig.
11	Pilgrimage is important to me.	Pilgern ist mir wichtig.
12	Pilgrimage has a high priority in my life.	Pilgern hat einen hohen Stellenwert in meinem Leben.
13	It is important to me to be good at pilgrimage.	Es ist mir wichtig, gut im Pilgern zu sein.

Note. Answer format for the English items ranges from 1 (*completely disagree*) to 5 (*completely agree*). Answer format for the German items ranges from 1 (*stimmt gar nicht*) to 5 (*stimmt genau*).

SM4: Assessments of Motivation

The German version of the items was used in this study. The English version has been translated via artificial intelligence (deepl.com) and has not yet been psychometrically evaluated.

SM4_1: ***Motivation Assessment – Study 1 (Yoga-Trait)***

Nr.	English	German
1	I am usually motivated to practise yoga.	Ich bin üblicherweise motiviert Yoga zu praktizieren.

Note. Answer format for the English items ranges from 1 (*completely disagree*) to 5 (*completely agree*). Answer format for the German items ranges from 1 (*stimmt gar nicht*) to 5 (*stimmt genau*).

SM4_2: ***Motivation Assessment – Study 2 (Yoga-State)***

Nr.	English	German
1	I am usually motivated to practise yoga.	Ich bin üblicherweise motiviert Yoga zu praktizieren.

Note. Answer format for the English items ranges from 1 (*completely disagree*) to 5 (*completely agree*). Answer format for the German items ranges from 1 (*stimmt gar nicht*) to 5 (*stimmt genau*).

SM4_3: ***Motivation Assessment – Study 3 (Meditation-Trait)***

Nr.	English	German
1	I am ususally motivated to meditate.	Ich bin üblicherweise motiviert zu meditieren.

Note. Answer format for the English items ranges from 1 (*completely disagree*) to 5 (*completely agree*). Answer format for the German items ranges from 1 (*stimmt gar nicht*) to 5 (*stimmt genau*).

SM4_4: ***Motivation Assessment – Study 4 (Meditation-State)***

Nr.	English	German
1	I am usually motivated to meditate.	Ich bin üblicherweise motiviert zu meditieren.

Note. Answer format for the English items ranges from 1 (*completely disagree*) to 5 (*completely agree*). Answer format for the German items ranges from 1 (*stimmt gar nicht*) to 5 (*stimmt genau*).

SM4_5: ***Motivation Assessment – Study 5 (Silence Retreat-Trait)***

**Nr.**	**English**	**German**
1	I am usually motivated to take part in silence retreats.	Ich bin üblicherweise motiviert, an Schweigeretreats teilzunehmen.
2	I am looking forward to taking part in my next silence retreat.	Ich freue mich auf meine Teilnahme am nächsten Schweigeretreat.
3	I like to use my vacation to participate in silence retreats.	Ich nutze gerne meinen Urlaub, um an Schweigeretreats teilzunehmen.
4	When I don't take part in silence retreats, I miss it.	Wenn ich nicht bei Schweigeretreats teilnehme, dann vermisse ich es.
5	I usually feel like taking part in silence retreats.	Ich habe üblicherweise Lust, an Schweigeretreats teilzunehmen.

Note. Answer format for the English items ranges from 1 (*completely disagree*) to 5 (*completely agree*). Answer format for the German items ranges from 1 (*stimmt gar nicht*) to 5 (*stimmt genau*).

SM4_6: ***Motivation Assessment – Study 6 (Silence Retreat-State)***

Nr.	English	German
1	I am usually motivated to take part in silence retreats.	Ich bin üblicherweise motiviert, an Schweigeretreats teilzunehmen.
2	I am looking forward to taking part in my next silence retreat.	Ich freue mich auf meine Teilnahme am nächsten Schweigeretreat.
3	I like to use my vacation to participate in silence retreats.	Ich nutze gerne meinen Urlaub, um an Schweigeretreats teilzunehmen.
4	When I don't take part in silence retreats, I miss it.	Wenn ich nicht bei Schweigeretreats teilnehme, dann vermisse ich es.
5	I usually feel like taking part in silence retreats.	Ich habe üblicherweise Lust, an Schweigeretreats teilzunehmen.

Note. Answer format for the English items ranges from 1 (*completely disagree*) to 5 (*completely agree*). Answer format for the German items ranges from 1 (*stimmt gar nicht*) to 5 (*stimmt genau*).

SM4_7: ***Motivation Assessment – Study 7 (Sermon-Trait)***

Nr.	English	German
1	I go to church services because the sermon interests me.	Ich gehe zum Gottesdienst, mich die Predigt interessiert.
2	I go to church services because the sermon offers me guidance in everyday life.	Ich gehe zum Gottesdienst, da mir die Predigt Orientierung im Alltag bietet.

Note. Answer format for the English items ranges from 1 (*completely disagree*) to 5 (*completely agree*). Answer format for the German items ranges from 1 (*stimmt gar nicht*) to 5 (*stimmt genau*).

SM4_8: ***Motivation Assessment – Study 8 (Sermon-State)***

Nr.	English	German
1	I go to church services because the sermon interests me.	Ich besuche den Gottesdienst, weil mir die Predigt Orientierung im Alltag bietet.
2	I go to church services because the sermon offers me guidance in everyday life.	Ich besuche den Gottesdienst, weil mich der Inhalt der Predigt interessiert.

Note. Answer format for the English items ranges from 1 (*completely disagree*) to 5 (*completely agree*). Answer format for the German items ranges from 1 (*stimmt gar nicht*) to 5 (*stimmt genau*).

SM4_9: ***Motivation Assessment – Study 9 (Pilgrimage-Trait)***

Nr.	English	German
1	I am usually motivated to go on pilgrimages.	Ich bin üblicherweise motiviert zu pilgern.
2	I am looking forward to going on the next pilgrimage.	Ich freue mich darauf, auf die nächste Pilgerreise zu gehen.
3	I usually feel like going on a pilgrimage.	Ich habe üblicherweise Lust auf Pilgern.
4	If I don't go on a pilgrimage, I miss it.	Wenn ich nicht pilgere, vermisse ich es.
5	I like to use my vacation to go on pilgrimages.	Ich nutze gerne meinen Urlaub, um zu pilgern.

Note. Answer format for the English items ranges from 1 (*completely disagree*) to 5 (*completely agree*). Answer format for the German items ranges from 1 (*stimmt gar nicht*) to 5 (*stimmt genau*).

SM4_10: ***Motivation Assessment – Study 10 (Pilgrimage-State)***

Nr.	English	German
1	I am usually motivated to go on pilgrimages.	Ich bin üblicherweise motiviert zu pilgern.
2	I am looking forward to going on the next pilgrimage.	Ich freue mich darauf, auf die nächste Pilgerreise zu gehen.
3	I usually feel like going on a pilgrimage.	Ich habe üblicherweise Lust auf Pilgern.
4	If I don't go on a pilgrimage, I miss it.	Wenn ich nicht pilgere, vermisse ich es.
5	I like to use my vacation to go on pilgrimages.	Ich nutze gerne meinen Urlaub, um zu pilgern.

Note. Answer format for the English items ranges from 1 (*completely disagree*) to 5 (*completely agree*). Answer format for the German items ranges from 1 (*stimmt gar nicht*) to 5 (*stimmt genau*).

SM5: ***Percentages of Boredom Single-Item Indicator and Scale Values Above Given Thresholds***

Study	Trait/State	3 to 5	4 to 5	5
		Scale		
Yoga	Trait	45.96	10.56	0.62
Yoga	State	15.79	8.77	0.00
Meditation	Trait	60.32	15.87	3.17
Meditation	State	11.48	3.28	0.00
Silence retreat	Trait	20.73	3.66	0.00
Silence retreat	State	19.05	4.76	2.38
Sermon	Trait	92.74	69.83	30.45
Sermon	State	36.08	16.49	5.15
Pilgrimage	Trait	11.11	0.00	0.00
Pilgrimage	State	8.06	0.00	0.00
		Single-item		
Yoga	Trait	19.88	6.83	1.24
Yoga	State	10.53	7.02	0.00
Meditation	Trait	48.39	22.58	1.61
Meditation	State	14.75	0.00	0.00
Silence retreat	Trait	9.76	3.66	1.22
Silence retreat	State	21.43	7.14	4.76
Sermon	Trait	83.15	54.78	26.69
Sermon	State	20.62	17.53	7.22
Pilgrimage	Trait	5.56	0.00	0.00
Pilgrimage	State	4.03	0.00	0.00

Note. Values are provided as percentages.

SM6: Correlations Between Scales

SM6_1: ***Correlations Among Variables – Study 1 (Yoga-Trait)***

Yoga-related variables	1.	2.	3.	4.	5.
1. Trait boredom					
2. Overchallenge	.44 ***				
3. Underchallenge	.54 ***	.37 ***			
4. Value	-.62 ***	-.26 ***	-.25 **		
5. Motivation	-.49 ***	-.31 ***	-.31 ***	.59 ***	
M (*SD*)	2.16 (0.66)	2.11 (0.74)	1.90 (0.77)	2.98 (0.71)	3.51 (1.03)

Note. $N = 159$. All constructs were assessed by using 5-point rating scales ranging from 1 (*completely disagree*) to 5 (*completely agree*). *) $p < .05$; **) $p < .01$; ***) $p < .001$.

SM6_2: ***Correlations Among Variables – Study 2 (Yoga-State)***

Yoga-related variables	1.	2.	3.	4.	5.
1. State boredom					
2. Overchallenge	.29 *				
3. Underchallenge	.72 ***	.46 ***			
4. Value	-.56 ***	-.26 +	-.52 ***		
5. Motivation	-.66 ***	-.26 +	-.56 ***	.60 ***	
M (*SD*)	1.42 (0.68)	1.80 (0.60)	1.73 (0.72)	3.57 (0.54)	4.28 (0.86)

Note. $N = 57$. All constructs were assessed by using a 5-point rating scale ranging from 1 (*completely disagree*) to 5 (*completely agree*). *) $p < .05$; **) $p < .01$; ***) $p < .001$.

SM6_3: ***Correlations Among Variables – Study 3 (Meditation-Trait)***

Meditation-related variables	1.	2.	3.	4.	5.
1. Trait boredom					
2. Overchallenge	.36 **				
3. Underchallenge	.48 ***	.55 ***			
4. Value	-.56 ***	-.07	-.10		
5. Motivation	-.36 +	-.28	-.53 **	.39 *	
M (*SD*)	2.39 (0.83)	2.05 (0.84)	2.04 (0.89)	2.79 (0.61)	3.52 (0.83)

Note. N range: 28 to 63. All constructs were assessed by using a 5-point rating scale ranging from 1 (*completely disagree*) to 5 (*completely agree*). +) $p < .10$; *) $p < .05$; **) $p < .01$; ***) $p < .001$.

SM6_4: ***Correlations among variables – Study 4 (Meditation-State)***

Meditation-related variables	1.	2.	3.	4.	5.
1. State boredom					
2. Overchallenge	.35 **				
3. Underchallenge	.42 ***	.60 ***			
4. Value	-.28 *	-.29 *	-.10		
5. Motivation	-.28 *	-.26 *	-.27 *	.45 ***	
M (*SD*)	1.48 (0.59)	1.50 (0.63)	1.50 (0.66)	3.54 (0.51)	3.61 (1.02)

Note. N range: 59 to 61. All constructs were assessed by using a 5-point rating scale ranging from 1 (*completely disagree*) to 5 (*completely agree*). *) $p < .05$; **) $p < .01$; ***) $p < .001$.

SM6_5: ***Correlations among variables – Study 5 (Silence retreat - Trait)***

Silence retreat-related variables	1.	2.	3.	4.	5.
1. Trait boredom					
2. Overchallenge	.45 ***				
3. Underchallenge	.29 **	.37 ***			
4. Value	-.53 ***	-.27 *	-.32 **		
5. Motivation	-.47 ***	-.30 **	-.18	.69 ***	
M (*SD*)	1.63 (0.60)	1.91 (0.52)	1.75 (0.78)	3.66 (0.70)	3.60 (0.94)

Note. N range: 77 to 80. All constructs were assessed by using a 5-point rating scale ranging from 1 (*completely disagree*) to 5 (*completely agree*). *) $p < .05$; **) $p < .01$; ***) $p < .001$.

SM6_6: ***Correlations Among Variables – Study 6 (Silence Retreat-State)***

Silence retreat-related variables	1.	2.	3.	4.	5.
1. State boredom					
2. Overchallenge	.62 **				
3. Underchallenge	.58 **	.85 ***			
4. Value	-.68 **	-.37	-.58 **		
5. Motivation	-.64 **	-.37	-.32	.56 *	
M (*SD*)	1.72 (0.93)	1.63 (0.46)	1.59 (0.63)	3.96 (0.72)	3.61 (1.20)

Note. N range: 19 to 20. All constructs were assessed by using a 5-point rating scale ranging from 1 (*completely disagree*) to 5 (*completely agree*). *) $p < .05$; **) $p < .01$; ***) $p < .001$.

SM6_7: ***Correlations Among Variables – Study 7 (Sermon-Trait)***

Sermon-related variables	1.	2.	3.	4.	5.
1. Trait boredom					
2. Overchallenge	.03				
3. Underchallenge	.23 ***	-.14 **			
4. Value	-.68 ***	.07	-.15 **		
5. Motivation	-.58 ***	.12 *	-.16 **	.72 ***	
M (*SD*)	3.56 (0.94)	1.80 (0.97)	2.90 (1.21)	1.80 (0.80)	1.77 (0.95)

Note. N range: 346 to 355. All constructs were assessed by using a 5-point rating scale ranging from 1 (*completely disagree*) to 5 (*completely agree*). *) $p < .05$; **) $p < .01$; ***) $p < .001$.

SM6_8: ***Correlations Among Variables – Study 8 (Sermon-State)***

Sermon-related variables	1.	2.	3.	4.	5.
1. State boredom					
2. Overchallenge	.75 ***				
3. Underchallenge	.56 ***	.66 ***			
4. Value	-.39 ***	-.29 **	-.22 *		
5. Motivation	-.23 *	.01	.01	.56 ***	
M (*SD*)	1.98 (1.09)	1.79 (1.33)	2.02 (1.24)	3.30 (0.92)	3.52 (0.99)

Note. N range: 95 to 97. All constructs were assessed by using a 5-point rating scale ranging from 1 (*completely disagree*) to 5 (*completely agree*). *) $p < .05$; **) $p < .01$; ***) $p < .001$.

SM6_9: ***Correlations Among Variables – Study 9 (Pilgrimage-Trait)***

Pilgrimage-related variables	1.	2.	3.	4.	5.
1. Trait boredom					
2. Overchallenge	.53 ***				
3. Underchallenge	.41 ***	.35 ***			
4. Value	-.59 ***	-.30 ***	-.23 **		
5. Motivation	-.53 ***	-.30 ***	-.25 **	.60 ***	
M (*SD*)	1.44 (0.43)	1.71 (0.47)	1.90 (0.56)	3.93 (0.46)	4.36 (0.75)

Note. $N = 162$. All constructs were assessed by using a 5-point rating scale ranging from 1 (*completely disagree*) to 5 (*completely agree*). *) $p < .05$; **) $p < .01$; ***) $p < .001$.

SM6_10: ***Correlations Among Variables – Study 10 (Pilgrimage-State)***

Pilgrimage-related variables	1.	2.	3.	4.	5.
1. State boredom					
2. Overchallenge	.50 ***				
3. Underchallenge	.34 ***	.29 ***			
4. Value	-.37 ***	-.23 **	-.22 *		
5. Motivation: general intensity	-.33 ***	-.22 *	-.05	.59 ***	
M (SD)	1.35 (0.43)	1.75 (0.50)	1.82 (0.48)	3.89 (0.49)	4.12 (0.80)

Note. N = 124. All constructs were assessed by using a 5-point rating scale ranging from 1 (*completely disagree*) to 5 (*completely agree*). *) $p < .05$; **) $p < .01$; ***) $p < .001$.

SM7: Meta-analytic findings

SM7_1: ***Single-Mean Meta-Analyses of Spiritual Boredom Across Studies – Scale Means***

Model	*k*	Summary mean	I^2	*Q*
Overall	10	1.91 [1.48; 2.33]	1.00	1821.35***
Trait	5	2.24 [1.50; 2.97]	1.00	1459.23***
State	5	1.56 [1.35; 1.77]	0.87	31.63***

Note. ***) $p < .001$. *k* indicates the number of included studies. I^2 denotes the percentage of variance explained by between-study heterogeneity relative to sampling variance, whereas *Q* is a parameter used to quantify between-study heterogeneity (Borenstein et al., 2021).

SM7_2: ***Single-Mean Meta-Analyses of Spiritual Boredom Across Studies – Single-Item Means***

Model	*k*	Summary mean	I^2	*Q*
Overall	10	1.88 [1.44; 2.33]	0.99	1371.79***
Trait	5	2.18 [1.36; 2.99]	1.00	1104.69***
State	5	1.56 [1.33; 1.80]	0.87	31.92***

Note. ***) $p < .001$. *k* indicates the number of included studies. I^2 denotes the percentage of variance explained by between-study heterogeneity relative to sampling variance, whereas *Q* is a parameter used to quantify between-study heterogeneity (Borenstein et al., 2021).

SM7_3: ***Mixed-Effects Meta-Analyses of Correlations with Spiritual Boredom, Moderation by Trait vs. State***

Model	β	*k*	I^2	*Q*
Overchallenge	0.19 [-0.13; 0.48]	10	84.93	68.24***
Underchallenge	0.16 [-0.08; 0.39]	10	71.92	30.10***
Value	0.27*** [0.12; 0.41]	10	30.23	12.30
Motivation	0.14 [-0.06; 0.33]	10	57.12	17.63*

Note. *) $p < .05$; **) $p < .01$; ***) $p < .001$. *k* indicates the number of included studies. I^2 denotes the percentage of variance explained by between-study heterogeneity relative to sampling variance, whereas *Q* is a parameter used to quantify between-study heterogeneity (Borenstein et al., 2021).

SM7_4: ***Meta-Analyses of Correlations with Spiritual Boredom, Subgroups by Trait vs. State***

Model	Trait vs. State	Summary *r*	*k*	I^2	*Q*
Overchallenge	Trait	.37*** [.16; .54]	10	84.93	68.24***
Overchallenge	State	.52*** [.33; .68]	10	84.93	68.24***
Underchallenge	Trait	.39*** [.25; .52]	10	71.92	30.10***
Underchallenge	State	.52*** [.38; .64]	10	71.92	30.10***
Value	Trait	-.62*** [-.67; -.56]	10	30.23	12.30
Value	State	-.42*** [-.51; -.31]	10	30.23	12.30
Motivation	Trait	-.51*** [-.60; -.41]	10	57.12	17.63*
Motivation	State	-.40*** [-.52; -.27]	10	57.12	17.63*

Note. *) $p < .05$; **) $p < .01$; ***) $p < .001$. *k* indicates the number of included studies. I^2 denotes the percentage of variance explained by between-study heterogeneity relative to sampling variance, whereas *Q* is a parameter used to quantify between-study heterogeneity (Borenstein et al., 2021).

SM8: ***Descriptive Statistics on Participants' Current Spiritual Practices***

Spiritual Context	Study	Trait/State	Variable	*M*	*SD*
Yoga	1	Trait	[a]Frequency of yoga practice	1.92	0.83
	2	State	[b]Frequency of yoga practice	1.72	0.45
Meditation	3	Trait	[a]Frequency of meditation	1.53	0.64
	4	State	[b]Frequency of meditation	1.48	0.50
Silence retreat	5	Trait	No. of previous silence retreats	6.90	6.40
	6	State	No. of previous silence retreats	14.21	27.25
Sermon	7	Trait	[c]Frequency of service attendance	1.32	0.64
	8	State	[c]Frequency of service attendance	2.54	0.69
Pilgrimage	9	Trait	No. of previous pilgrimages	5.99	4.03
	10	State	No. of previous pilgrimages	7.70	8.51

Note. a) Participants responded how often they practiced yoga/meditation on a scale ranging from 1 to 3 (1 = *yes, regularly*; 2 = *yes, but not regularly*; 3 = *not currently*). b) Participants responded how often they practiced yoga/meditation on a scale ranging from 1 to 2 (1 = *yes, regularly*; 2 = *yes, but not regularly*). c) Participants responded how often they attended church services on a scale ranging from 1 to 3 (1 = *once every week*; 2 = *multiple times per year, but less than once every week*; 3 = *once per year or less*).
