# Supplementary material for: Spiritual boredom is associated with over- and underchallenge, lack of value, and reduced motivation
Source: Commun Psychol. 2025 Mar 5;3:35. doi: 10.1038/s44271-025-00216-7 (PMC11882887; doi:10.1038/s44271-025-00216-7)
Supplement: Supplementary file 3 — Reporting Summary [file 44271_2025_216_MOESM3_ESM.pdf]

nature portfolio

Corresponding author(s): Thomas Goetz / COMMSPSYCHOL-24-0597

Last updated by author(s): Feb 3, 2025

Reporting Summary

Nature Portfolio wishes to improve the reproducibility of the work that we publish. This form provides structure for consistency and transparency in reporting. For further information on Nature Portfolio policies, see our Editorial Policies and the Editorial Policy Checklist.

Statistics

For all statistical analyses, confirm that the following items are present in the figure legend, table legend, main text, or Methods section.

n/a	Confirmed	
☐	☒	The exact sample size (*n*) for each experimental group/condition, given as a discrete number and unit of measurement
☐	☒	A statement on whether measurements were taken from distinct samples or whether the same sample was measured repeatedly
☐	☒	The statistical test(s) used AND whether they are one- or two-sided<br>*Only common tests should be described solely by name; describe more complex techniques in the Methods section.*
☒	☐	A description of all covariates tested
☒	☐	A description of any assumptions or corrections, such as tests of normality and adjustment for multiple comparisons
☐	☒	A full description of the statistical parameters including central tendency (e.g. means) or other basic estimates (e.g. regression coefficient) AND variation (e.g. standard deviation) or associated estimates of uncertainty (e.g. confidence intervals)
☐	☒	For null hypothesis testing, the test statistic (e.g. *F*, *t*, *r*) with confidence intervals, effect sizes, degrees of freedom and *P* value noted<br>*Give P values as exact values whenever suitable.*
☒	☐	For Bayesian analysis, information on the choice of priors and Markov chain Monte Carlo settings
☒	☐	For hierarchical and complex designs, identification of the appropriate level for tests and full reporting of outcomes
☐	☒	Estimates of effect sizes (e.g. Cohen's *d*, Pearson's *r*), indicating how they were calculated

Our web collection on statistics for biologists contains articles on many of the points above.

Software and code

Policy information about availability of computer code

Data collection	The SoSci Survey platform (Leiner, 2024) was used for the online assessments.
Data analysis	All statistical analyses were conducted in R 4.4.2 (R Core Team, 2022). The R package meta was used to calculate the SMMAs (Balduzzi et al., 2019). For meta-analyses of effect sizes, we used the metafor package (Viechtbauer, 2010). For data visualization, we used the ggplot2 package (Wickham, 2016).

For manuscripts utilizing custom algorithms or software that are central to the research but not yet described in published literature, software must be made available to editors and reviewers. We strongly encourage code deposition in a community repository (e.g. GitHub). See the Nature Portfolio guidelines for submitting code & software for further information.

Data

Policy information about availability of data

All manuscripts must include a data availability statement. This statement should provide the following information, where applicable:

- Accession codes, unique identifiers, or web links for publicly available datasets
- A description of any restrictions on data availability
- For clinical datasets or third party data, please ensure that the statement adheres to our policy

All data, measures, and analysis codes are available at https://doi.org/10.17605/OSF.IO/G5PCU

Human research participants

Policy information about studies involving human research participants and Sex and Gender in Research.

Reporting on sex and gender	We assessed self-reported gender using the categories female/ male/diverse. Accordingly, we use the term 'gender' throughout our studies. As gender was not part of any of our hypotheses and was not used as a control variable, it was not included in any analyses. We provide disaggregated gender data in the source data.
Population characteristics	For all 10 studies reported in our manuscript, detailed population characteristics are presented in Table 1 of the manuscript. These characteristics were not used as covariates in our analyses.
Recruitment	All 10 studies took place between December 2021 and July 2024. For all studies, participants were recruited through a variety of methods, including using existing contacts with practitioners of the spiritual practice and institutions offering such practices (e.g., yoga studios, pilgrim associations), and social networks such as WhatsApp, LinkedIn, and Facebook. Contacted individuals and institutions were asked to share information about the study (i.e., snowball sampling; Parker et al., 2019), through which a link to an online questionnaire was distributed.
Ethics oversight	Institutional Review Board (IRB) of the Department of Developmental and Educational Psychology at the University of Vienna

Note that full information on the approval of the study protocol must also be provided in the manuscript.

Field-specific reporting

Please select the one below that is the best fit for your research. If you are not sure, read the appropriate sections before making your selection.

☐ Life sciences ☒ Behavioural & social sciences ☐ Ecological, evolutionary & environmental sciences

For a reference copy of the document with all sections, see nature.com/documents/nr-reporting-summary-flat.pdf

Behavioural & social sciences study design

All studies must disclose on these points even when the disclosure is negative.

Study description	quantitative, cross-sectional
Research sample	Individuals over the age of 18 with experience of spiritual practice were eligible to participate in the studies. Demographic data are detailed in Table 1 of our manuscript.
Sampling strategy	All 10 studies took place between December 2021 and July 2024. For all studies, participants were recruited through a variety of methods, including using existing contacts with practitioners of the spiritual practice and institutions offering such practices (e.g., yoga studios, pilgrim associations), and social networks such as WhatsApp, LinkedIn, and Facebook. Contacted individuals and institutions were asked to share information about the study (i.e., snowball sampling; Parker et al., 2019), through which a link to an online questionnaire was distributed.<br>For the trait assessments, we aimed to recruit participants with experience in the spiritual practice being studied (i.e., former and current practitioners). For the state assessments, we sought participants who were currently practicing the respective spiritual practice. The questionnaires were created using the SoSci Survey platform (Leiner, 2024). Upon activation of the link, participants received detailed information about the study, data handling procedures, guarantees of full anonymity, and contact information for the researchers. An exception was the study on state sermon boredom (Study 8). In this study, participants were recruited by trained test administrators in four Catholic churches directly after attending a service.<br>In all 10 studies, participants had to be at least 18 years old to take part. Participants had to provide their consent before proceeding with the questionnaire, which began with demographic information, followed by the assessment of all other variables. Participants could stop the assessment at any time without having to give a reason. The average time taken to complete the questionnaires across the 10 studies ranged from 2.97 minutes (silence retreat – trait; Study 6) to 8.08 minutes (pilgrimage – state; Study 10).
Data collection	Both online and paper-pencil assessments were used. All measures from all 10 studies are presented in the online supplemental material.
Timing	All 10 studies took place between December 2021 and July 2024.
Data exclusions	No data were excluded.
Non-participation	It was a cross-sectional design - no participants dropped out.
Randomization	Participants were not allocated into experimental groups.

Reporting for specific materials, systems and methods

We require information from authors about some types of materials, experimental systems and methods used in many studies. Here, indicate whether each material, system or method listed is relevant to your study. If you are not sure if a list item applies to your research, read the appropriate section before selecting a response.

Materials & experimental systems

n/a	Involved in the study
☒	☐ Antibodies
☒	☐ Eukaryotic cell lines
☒	☐ Palaeontology and archaeology
☒	☐ Animals and other organisms
☒	☐ Clinical data
☒	☐ Dual use research of concern

Methods

n/a	Involved in the study
☒	☐ ChIP-seq
☒	☐ Flow cytometry
☒	☐ MRI-based neuroimaging
